# Trypanosoma brucei Tim50 Possesses PAP Activity and Plays a Critical Role in Cell Cycle Regulation and Parasite Infectivity

**DOI:** 10.1128/mBio.01592-21

**Published:** 2021-09-14

**Authors:** Anuj Tripathi, Ujjal K. Singha, Ayorinde Cooley, Taneisha Gillyard, Evan Krystofiak, Siddharth Pratap, Jamaine Davis, Minu Chaudhuri

**Affiliations:** a Department of Microbiology, Immunology, and Physiology, School of Medicine, Meharry Medical College, Nashville, Tennessee, USA; b Department of Cell and Developmental Biology, Vanderbilt University, Nashville, Tennessee, USA; University of California Los Angeles

**Keywords:** infectivity, protein translocase, *Trypanosoma brucei*, cell cycle, kDNA, mitochondria

## Abstract

Trypanosoma brucei, the infective agent for African trypanosomiasis, possesses a homologue of the translocase of the mitochondrial inner membrane 50 (TbTim50). It has a pair of characteristic phosphatase signature motifs, DXDX(T/V). Here, we demonstrated that, besides its protein phosphatase activity, the recombinant TbTim50 binds and hydrolyzes phosphatidic acid in a concentration-dependent manner. Mutations of D^242^ and D^244^, but not of D^345^and D^347^, to alanine abolished these activities. *In silico* structural homology models identified the putative binding interfaces that may accommodate different phosphosubstrates. Interestingly, TbTim50 depletion in the bloodstream form (BF) of T. brucei reduced cardiolipin (CL) levels and decreased mitochondrial membrane potential (ΔΨ). TbTim50 knockdown (KD) also reduced the population of G_2_/M phase and increased that of G_1_ phase cells; inhibited segregation and caused overreplication of kinetoplast DNA (kDNA), and reduced BF cell growth. Depletion of TbTim50 increased the levels of AMPK phosphorylation, and parasite morphology was changed with upregulation of expression of a few stumpy marker genes. Importantly, we observed that TbTim50-depleted parasites were unable to establish infection in mice. Proteomics analysis showed reductions in levels of the translation factors, flagellar transport proteins, and many proteasomal subunits, including those of the mitochondrial heat shock locus ATPase (HslVU), which is known to play a role in regulation of kinetoplast DNA (kDNA) replication. Reduction of the level of HslV in TbTim50 KD cells was further validated by immunoblot analysis. Together, our results showed that TbTim50 is essential for mitochondrial function, regulation of kDNA replication, and the cell cycle in the BF. Therefore, TbTim50 is an important target for structure-based drug design to combat African trypanosomiasis.

## INTRODUCTION

Trypanosoma brucei is a parasitic protozoan and the infectious agent of a fatal disease in human and domestic animals, known as African trypanosomiasis (1). The disease is transmitted by the bite of the tsetse fly, which is prevalent in sub-Saharan Africa. In mammalian blood, T. brucei exists as a proliferative long-slender (LS) bloodstream form (BF) (2, 3). The LS form is covered with a thick surface coat consisting of a variant surface glycoprotein that periodically changes and protects the parasite from the host’s immune attack (4). At the peak of each parasitic wave, the LS form is differentiated to the nondividing stumpy (ST) form by a cell density-sensing phenomenon. The current understanding articulates that oligopeptides generated by the peptidases released from the LS form trigger an autocrine signaling mechanism via activation of the AMP-activated protein kinase (AMPK) and increase the expression of the ST-specific genes (5, 6). The gene expression pattern of each of these developmental forms has been widely investigated in T. brucei, and the signaling mechanisms involved in these processes have been gradually identified (7, 8).

T. brucei possesses a concatenated structure of mitochondrial DNA known as kinetoplast DNA (kDNA) that consists of thousands of minicircular and few dozen maxicircular DNAs. The kDNA disc is attached to the flagellar basal body through the mitochondrial membrane via a filamentous structure known as the tripartite attachment complex (TAC) (9, 10). kDNA plays a crucial role during cell division (11). kDNA duplication and segregation occur before nuclear duplication and division. Duplicated kDNA, basal body, flagella, and nucleus are separated into two daughter cells during cytokinesis, along with the division of single mitochondrion (11, 12). Despite its complex structure, mitochondrial DNA in T. brucei only encodes 18 proteins. Therefore, similarly to other eukaryotes, a vast majority of mitochondrial proteins are encoded in the nuclear genome and are imported into mitochondria after synthesis in the cytosol (13, 14).

Mitochondrial protein import machinery is conserved overall among fungi and animals. Three major complexes are present, namely, the translocase of the mitochondrial outer membrane (TOM) and two translocases of the mitochondrial inner membrane TIM23 and TIM22 (15, 16). Nuclear DNA-encoded mitochondrial proteins with either an N-terminal or an internal targeting signal cross the outer membrane (OM) through the TOM complex (17) and select either TIM23 or TIM22 complexes, respectively, to reach the destination (18, 19). The core components of the TIM23 complex are Tim17, Tim23, and Tim50 (20, 21). The first two components each have four transmembrane domains (TMDs) that form the import channel; Tim50 has a single TMD with a large C-terminal domain exposed in the intermembrane space (IMS) that acts as the receptor for the preprotein (22, 23). The T. brucei mitochondrial protein import machinery is significantly divergent. Instead of two TIM complexes, T. brucei likely possesses a single TIM (13, 14). The major component of the T. brucei TIM (TbTIM) is TbTim17 (24). Several other trypanosome-specific proteins are found associated with TbTim17, including TbTim62, TbTim42, TbTim54, two rhomboid-like proteins, acetyl coenzyme A (CoA) dehydrogenase, six small TbTims, and TbTim50, which is relatively conserved (24–29). The functions of these proteins have not been explored fully.

We identified the Tim50 homologue in T. brucei (TbTim50) and showed that it is involved in the import of preproteins into mitochondria (25). Like its homologues in other eukaryotes, TbTim50 possesses a characteristic C-terminal domain (CTD) containing a pair of DXDX(T/V) signature motifs, (^242^DLDET^246^ and ^345^DLDRV^349^) (25). A similar motif is found in the class of CTD phosphatases (Sc-FCP1/h-SCP1) that dephosphorylate the serine residues in the tail of the RNA polymerase II large subunit (30). The members of these CTD phosphatases are also involved in other cellular functions, such as regulation of proteasomal activity, stress tolerance, and dephosphorylation of different signaling factors (31, 32). Together, these proteins belong to a large superfamily known as the haloacid dehalogenases (HAD), which are universally present in both prokaryotes and eukaryotes (33). Substrates for HAD phosphatases differ widely and include small metabolites, proteins, and other macromolecules. Lipin, which hydrolyzes phosphatidic acid to diacyl glycerol and phosphate, also belongs to this family (34). Previously, we demonstrated that the recombinant TbTim50 possesses a dual-specific protein phosphatase activity (25). Later, we demonstrated that TbTim50 downregulation in the procyclic form (PF) of T. brucei that dwells in the insect vector increased tolerance to oxidative stress by increasing the levels of the phospho-tyrosyl phosphatase-interacting protein, PIP39 (35, 36). PIP39 is a similar HAD family phosphatase but is localized in the glycosomes in PF (37, 38). Glycosomes are peroxisome-like organelles harboring primarily the glycolytic enzymes in trypanosomatids (39). The communication between these phosphatases is likely linked via AMPK phosphorylation due to decreased production of ATP in TbTim50 knockdown (KD) PF (38). Here, we investigate the role of TbTim50 in the BF, the infective form of the parasite. We found that TbTim50 depletion is more detrimental in the BF than the PF. TbTim50 KD caused mitochondrial dysfunction and reduced the levels of the mitochondrial proteasomal subunit HslVU, a master regulator of kDNA replication, and thus arrested cell growth both *in vitro* and *in vivo*. Therefore, TbTim50 is essential for the parasite’s survival in its mammalian host.

## RESULTS

### TbTim50 can accommodate phosphoepitope binding and possesses PA-phosphatase activity.

**FIG 1:**
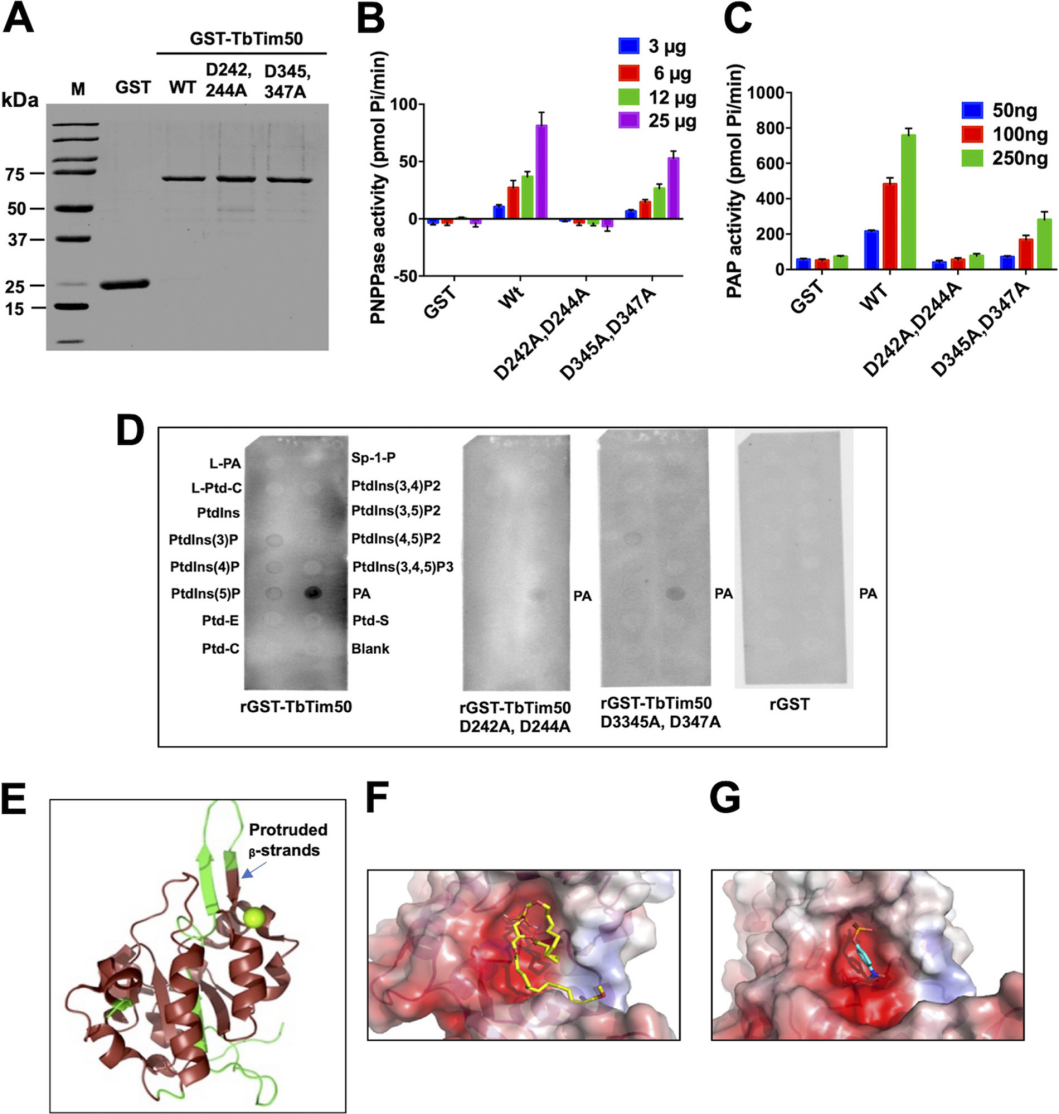
The purified recombinant TbTim50 binds and hydrolyzes phosphatidic acid (PA). (A) Recombinant glutathione *S*-transferase (GST)-TbTim50 (GST-TbTim50) wild type (WT), two mutants (D242-D244A and D345-D347A), and GST by itself were expressed in Escherichia coli and purified using affinity chromatography. (B) The phosphatase activity was measured using *para*-nitrophenyl phosphate (PNPP; 0.4 M) as the substrate and various amounts (3 to 25 μg) of purified recombinant proteins for 15 min. Released phosphate was quantitated by the fluorometric method as described in the additional material and methods in the supplemental materials (Text S1). (C) Recombinant TbTim50 displays phosphatidic acid phosphatase (PAP) activity. The enzymatic activity was measured by the release of phosphate from 1,2-dioctanoyl-*sn*-glycero-3-phosphate (DiC8 PA). The substrate was incubated with increasing amounts (50 to 250 μg) of proteins from either GST-TbTim50 WT, two mutants (D242-D244A and D345-D347A), or GST. The amount of phosphate released was measured by using PiBlue reagent and recording the absorbance at 620 nm. Error bars in panels B and C were calculated from three independent experiments. (D) rGST-TbTim50 binds with PA. A PIP Strip array was performed as described in Materials and Methods. The following spotted lipids are shown: L-PA, lyso-PA; L-Ptd-C, lyso-phosphatidyl choline; Ptdlns, phosphatidyl inositol; different Ptdlns-phosphates as indicated; Ptd-E, Ptd ethanolamine; Ptd-C, Ptd-choline; Sp-1-P, spingosine phosphate; PA, phosphatidic acid; and Ptd-S, Ptd serine. Purified recombinant proteins, GST-TbTim50 WT, two mutants (D242-D244A and D345-D347A), or GST were used as probes. (E) Structure homology modeling using the Cn3D program. The crystal structure of the ScTim50_IMS_-core region (PDB identifier 4QQF) was used as the template to compare to the predicted structure of TbTim50. (F) Molecular docking of phosphatidic acid (PA; Δ*G* = −4.8 kcal/mol) within the TbTim50 active site. (G) Molecular docking of *para*-nitrophenyl phosphate (PNPP; Δ*G* = −4.7 kcal/mol) within the TbTim50 active site.

We demonstrated previously that the recombinant TbTim50 (rTbTim50) could dephosphorylate both serine/threonine and tyrosine phospho-peptides, and that mutation of the conserved aspartate residues, D242 and D244, in TbTim50 abolished this activity (25). As a similar DXDX(T/V) motif was found in TbLipin (40, 41), we wanted to investigate whether TbTim50 also possesses phosphatidic acid (PA) phosphatase (PAP) activity. For this purpose, we purified the recombinant TbTim50 with a glutathione *S*-transferase (GST) tag (rGST-TbTim50) at the N terminus. The GST protein was purified in parallel to use as a negative control (Fig. 1A). In addition, we also generated two TbTim50 mutants, D242A-D244A and D345A-D347A by changing the conserved aspartic acid (D) residues within the DXDX(T/V) motifs to alanine (A) using a site-directed mutagenesis protocol as described in the additional materials and methods in the supplemental material (see Text S1 in the supplemental material). Mutated plasmids were sequenced for confirmation (Fig. S1A and B). The rGST-TbTim50 (wild type) hydrolyzed *para*-nitrophenyl phosphate (pNPP) in a dose-dependent manner, as reported previously (Fig. 1B). When PA was used as the substrate, rGST-TbTim50 also released phosphate in a dose-dependent manner (Fig. 1C), whereas rGST did not show any activity with either pNPP or PA (Fig. 1B and C). Mutation of D242 and D244 in TbTim50 to A abolished the phosphatase activities by almost 99% for both pNPP and PA. However, D345A and D347A mutations reduced activity by ∼50% and ∼75% for pNPP and PA, respectively (Fig. 1B and C). The PAP activity of the wild-type TbTim50 was 190 to 278 nmol/min/mg, whereas the PAP activities were 22 to 83 and 62 to 102 nmol/min/mg for TbTim50 mutants D242A-D244A and D345A-D347A, respectively (Table 1). Together, these results showed that TbTim50 phosphatase possesses a broader substrate specificity than was previously thought (25). Our results also showed that the first motif, ^242^DLDET^246^, is at the active site of the TbTim50 phosphatase and that the second motif, ^345^DLDRV^349^, plays a more auxiliary function, either to keep the correct conformation of the enzyme or to provide additional binding sites for different substrates.

**TABLE 1:** PAP activities of the TbTim50 wild type and mutants^*a*^

Protein	Activity (nmol/min/mg)
GST-TbTim50 (wild type)	234 ± 44
GST-TbTim50 D^242^A, D^244^A	52.5 ± 30.5
GST-TbTim50 D^345^A, D^347^A	82 ± 20
GST	46 ± 28

a PAP activity was measured using 1,2-dioctanoyl-*sn*-glycero-3-phosphate (DiC8 PA) as the substrate. Recombinant proteins were purified as described in the additional materials and methods in the supplemental material (Text S1). Specific activity was calculated from three independent experiments.

10.1128/mBio.01592-21.3 TEXT S1 Additional materials and methods. The corresponding references are included. Download Text S1, DOCX file, 0.03 MB. Copyright © 2021 Tripathi et al. 2021 Tripathi et al. https://creativecommons.org/licenses/by/4.0/ This content is distributed under the terms of the Creative Commons Attribution 4.0 International license.

10.1128/mBio.01592-21.4 FIG S1 Site-directed mutagenesis and structural homology model of TbTim50. Site-directed mutagenesis was performed using the QuikChange site-directed mutagenesis kit as described in the experimental procedures. Sequence for the mutated regions D242-D244 (A) and D345-D347 (B) are shown. As (marked as red) on the wild-type sequence are mutated to Cs. (C) The structural homology model of the C-terminal domain (CTD) PPase domain of TbTim50 (228 to 404 amino acids) highlighting the proximity of the active site D242 (red sphere) and the location of the predicted transmembrane segment (285 to 304 amino acids), shown as green helices. Download FIG S1, TIF file, 2.3 MB. Copyright © 2021 Tripathi et al. 2021 Tripathi et al. https://creativecommons.org/licenses/by/4.0/ This content is distributed under the terms of the Creative Commons Attribution 4.0 International license.

Next, we performed a lipid binding assay to check the binding affinity of TbTim50 with different phospholipids. A piece of nitrocellulose membrane containing 15 different phospholipid spots (100 pmol each) were probed with purified rGST-TbTim50 wild type and mutants, as well as with GST. Protein binding was visualized by anti-GST antibody and horseradish peroxidase (HRP)-conjugated secondary antibody. rGST-TbTim50 showed stronger binding affinity for PA, and no significant binding was observed with other lipids (Fig. 1D). In contrast to the wild type, the TbTim50 D242A-D244A and D345A-D347A mutants showed >90% and >50% reductions, respectively, in binding with PA. rGST showed no binding with any of these lipids. These results were correlated with the activity assay and further confirmed the importance of the first motif for PA binding and activity.

The active-site structures of several HAD phosphatases are well known (33). Furthermore, the crystal structure of the core domain of ScTbTim50 has also been published (42). Homology modeling of TbTim50 with the known structure of ScTim50 using the Cn3D program (https://www.ncbi.nlm.nih.gov/Structure/CN3D/cn3d.shtml) showed the presence of a similar core with multiple α-helices and β-sheets. However, the two antiparallel β-strands that protruded out of the core structure of ScTim50 did not match with the corresponding region in TbTim50 (Fig. 1E). In addition, TbTim50 is structurally distinct from other Tim50 homologues due to the presence of the transmembrane helix (285 to 310 amino acids [aa]) located within the CTD PPase domain (25) (Fig. S1C). To identify the putative binding pockets for the PA and pNPP substrates, we generated a homology model of the TbTim50 CTD PPase domain (228 to 404 aa) using the Phyre2 predictive modeling program (43). The model was validated using the SAVES server (https://saves.mbi.ucla.edu/) (Fig. S1C). Molecular docking analyses were conducted with both the PA and pNPP substrates using AutoDock Vina (Fig. 1F and G), and theoretical binding free energies were obtained (Table 2) (44). These results further confirmed that TbTim50 is a membrane-embedded enzyme capable of binding and hydrolyzing multiple substrates. These structural and docking predictions, coupled with TbTim50 possessing PAP activity, provide strong evidence that TbTim50 is indeed an HAD phosphatase.

**TABLE 2:** Top-ranked poses of the theoretical binding free energies evaluated through the AutoDock Vina scoring function^*a*^

Substrate^*c*^	Computed Δ*G* (kcal/mol)^*b*^
PA	−4.8
pNPP	−4.7

a Putative binding site residues were identified (using ChimeraX) as those interacting with the substrate molecule within 3.0 Å and were the same for both substrates, as follows: D242, D244, E245, S250, T299, A300, Y336, D354, N355, and S356. PA’s phosphate group interacts with Y336, while the phosphate group of pNPP interacts with D242.

b Δ*G*, binding free energy.

c PA, phosphatidic acid; pNPP, *para*-nitrophenyl phosphate.

### Tim50 is localized in mitochondria in the T. brucei BF.

**FIG 2:**
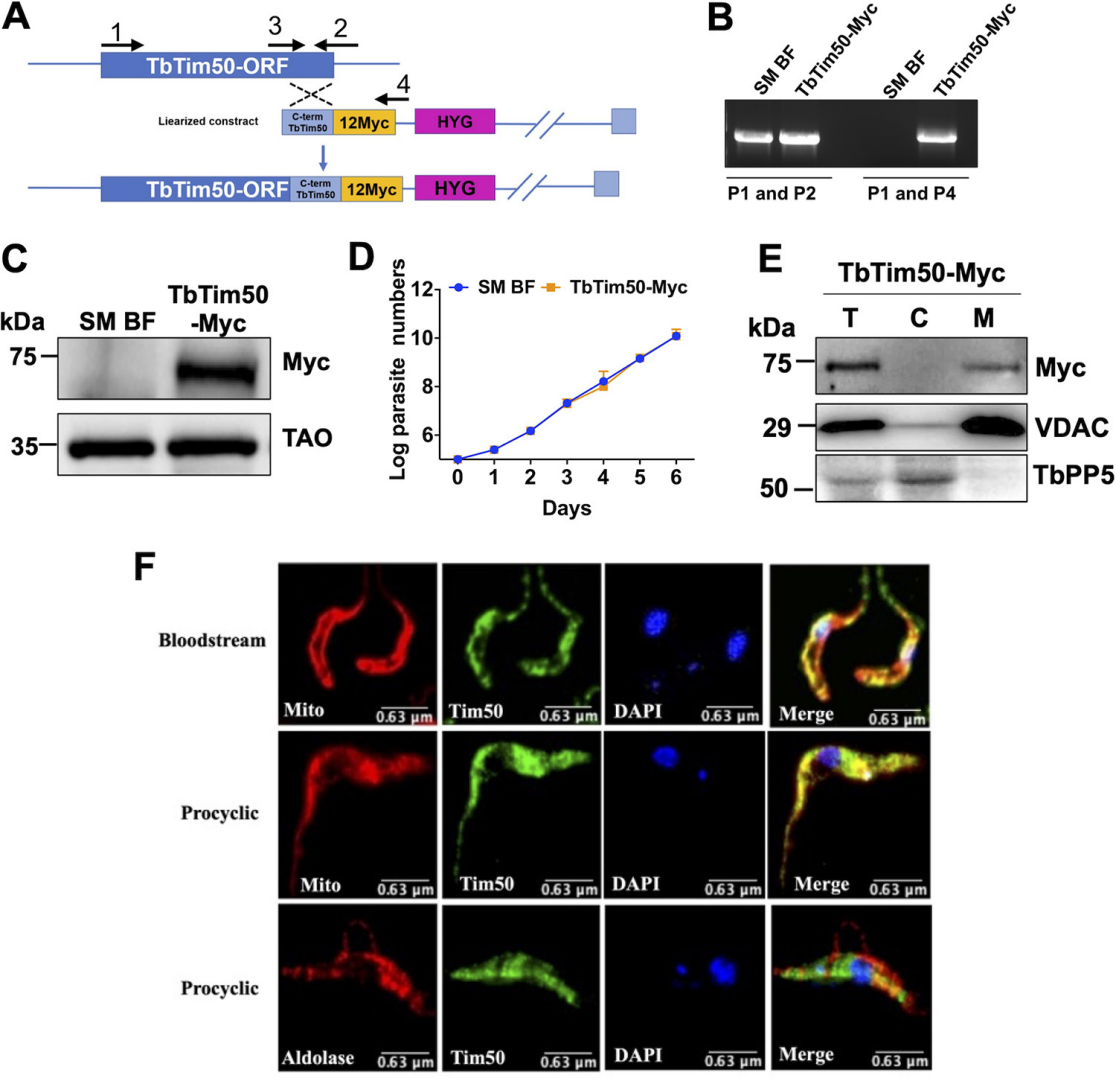
Expression and subcellular localization of the *in situ* Myc-tagged TbTim50 in T. brucei. (A) Schematic representation of the strategy used to generate *in situ*-tagged TbTim50-Myc construct. After transfection, the drug-selected parasites were cloned by limiting dilution. (B) Genomic PCR analysis of the unmodified and modified loci of TbTim50 was performed to verify the integration of the construct. Location of the primers used (P1 to P4) are shown in panel A. The amplicons were further sequenced for confirmation. (C) Immunoblot analysis of total proteins from the parental control (SM BF) and TbTim50-Myc cell lines probed with anti-Myc antibody. TAO was used as the loading control. (D) The SM BF and TbTim50-Myc cells were grown, and cell numbers were counted each day for 6 days. Cells were reinoculated when the parasite number reached 1 × 10^6^ cells/ml. The log of the cumulative cell number was plotted against the number of days postinduction. Standard errors were calculated from four independent experiments. (E) Total (“T”), cytosolic (“C”), and mitochondrial (“M”) fractions were collected after solubilization of the cell membrane with 0.03% digitonin, as described in Materials and Methods. Equal amounts of proteins (20 μg) were loaded per lane and immunoblotted with anti-Myc, anti-VDAC, and anti-TbPP5 antibodies. VDAC and TbPP5 were used for the mitochondrial and cytosolic markers, respectively. (F) *In situ* immunofluorescence staining of the TbTim50-Myc BF and PF. Live cells were stained with MitoTracker red, fixed, and stained with anti-Myc as the primary antibody and fluorescein isothiocyanate (FITC)-conjugated anti-mouse IgG as the secondary antibody. To localize glycosomal aldolase in the PF, anti-aldolase primary antibody and Texas-red-conjugated anti-rabbit secondary antibody were used. Images were taken by LSM 510 confocal microscope using ×60 magnification. Merged pictures are shown for colocalization.

Previously, we reported that TbTim50 is localized in mitochondria in PF (25). TbTim50 protein levels are similar in the PF and the ST BF, but relatively lower in the LS BF (45). To confirm the subcellular location of TbTim50 in the BF, we *in situ*-tagged TbTim50 with 12×Myc epitope at the C terminus (due to lower affinity of the available anti-TbTim50 antibody) using the vector pNATX12Myc and the strategy shown in Fig. 2A. Homologous recombination of the C terminus of the TbTim50 fragment with the endogenous locus was verified by genomic PCR analysis using gene-specific and vector-specific primers (Table S1). The gene-specific forward and reverse primers (P1 and P2) amplified the TbTim50 open reading frame (ORF) from both control and TbTim50-Myc transgenic cell lines. When the reverse primer was vector specific (P4), the expected-size product was found only from transfected cell DNA (Fig. 2B). Next, we checked the expression of TbTim50-Myc by immunoblot analysis. A protein band of around 75 kDa was detected by anti-Myc antibody from the stable transfected cell line (Tim50-Myc) but not from the single marker (SM) BF T. brucei (control) (Fig. 2C). The full gel picture is shown in Fig. S2. Expression of *in situ*-tagged TbTim50-Myc did not have any effect on cell growth (Fig. 2D). Cell fractionation analysis showed that TbTim50-Myc is present in the mitochondrial fraction and not in the cytosolic fraction. VDAC and TbPP5 were used as the mitochondrial and cytosolic markers (Fig. 2E). Immunofluorescence microscopy revealed that TbTim50-Myc, as stained by the anti-Myc primary and fluorescein isothiocyanate (FITC)-conjugated anti-mouse IgG secondary antibodies, did overlap MitoTracker-stained mitochondria in BF and PF cells that expressed TbTim50-Myc (Fig. 2F), whereas TbTim50-Myc stain did not overlapped with glycosomal protein aldolase (Fig. 2F). Therefore, as in the PF, TbTim50 is localized in mitochondria in the BF.

10.1128/mBio.01592-21.5 FIG S2 Expression of *in situ*-tagged TbTim50-Myc and the effect of TbTim50 RNAi. Immunoblot analysis of total proteins from the parental control (single marker blood form [SM BF]), TbTim50-Myc, and TbTim50-Myc/TbTim50 RNAi cells. The cell line TbTim50-Myc/TbTim50RNAi were grown in the presence of doxycycline, and cells were harvested at days 2 and 4 postinduction for analysis. (A) The blot was probed with anti-Myc antibody. (B) TAO was used as the loading control. The position of the specific band for TbTim50-Myc (∼75 kDa) is marked with an asterisk (*). Download FIG S2, TIF file, 1.4 MB. Copyright © 2021 Tripathi et al. 2021 Tripathi et al. https://creativecommons.org/licenses/by/4.0/ This content is distributed under the terms of the Creative Commons Attribution 4.0 International license.

10.1128/mBio.01592-21.10 TABLE S1 Primers used in this study. The forward (F) and reverse (R) primers used to generate various constructs are shown. Download Table S1, DOCX file, 0.02 MB. Copyright © 2021 Tripathi et al. 2021 Tripathi et al. https://creativecommons.org/licenses/by/4.0/ This content is distributed under the terms of the Creative Commons Attribution 4.0 International license.

### Tim50 knockdown in BF reduced cell growth and mitochondrial membrane potential.

**FIG 3:**
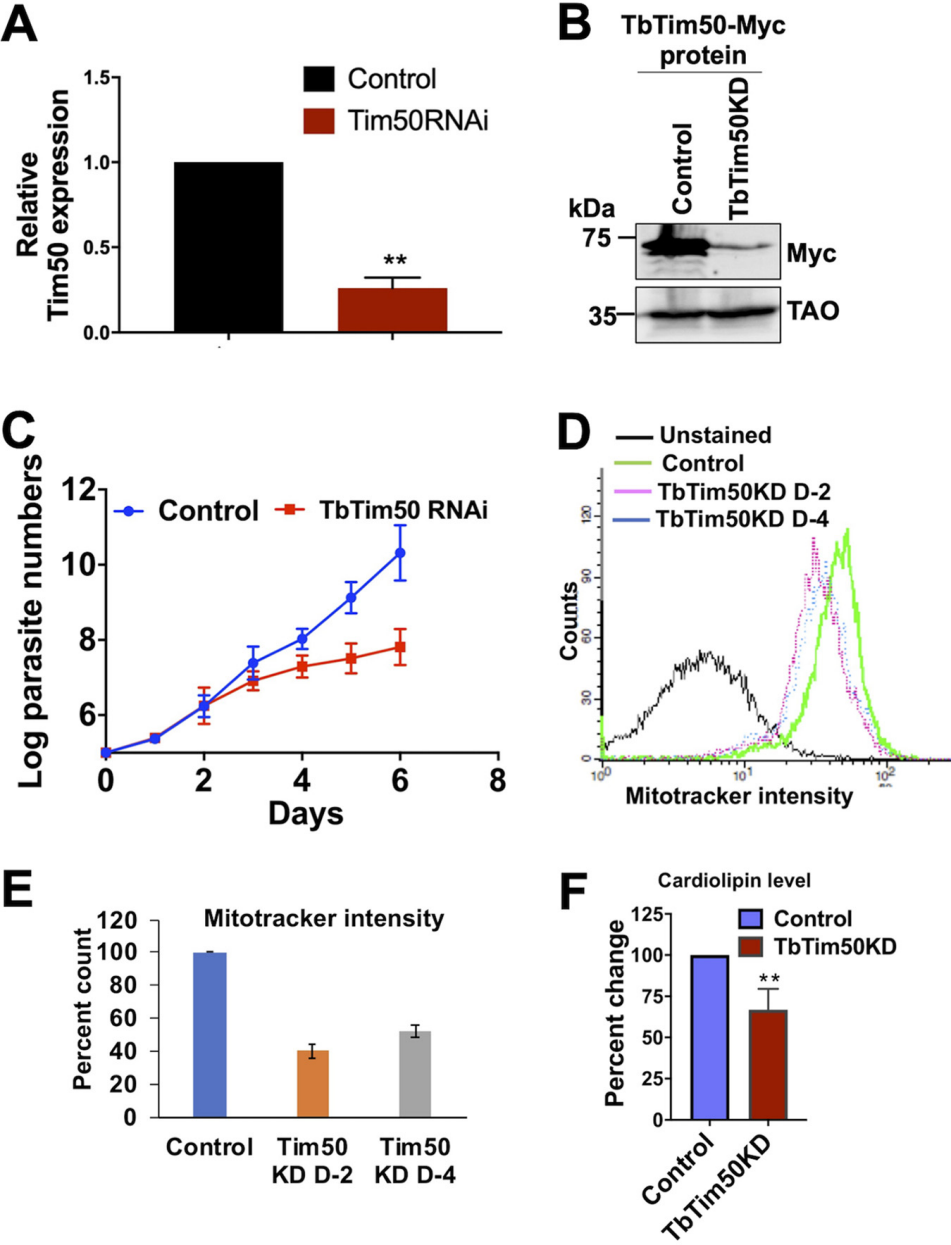
TbTim50 depletion inhibits BF cell growth and reduces cardiolipin levels and mitochondrial ΔΨ. (A) Reverse transcription-PCR (RT-PCR) analysis of the TbTim50 transcript levels in TbTim50-Myc (control) and TbTim50-Myc/TbTim50 RNA interference (RNAi) cells after 48 h of induction with doxycycline. The levels of the target transcript were normalized with the levels of tubulin transcript in each sample, and the normalized value for control was considered 100%. (B) Immunoblot analysis of total proteins from TbTim50-Myc (control) and TbTim50-Myc/TbTim50 RNAi (TbTim50 KD) cells grown in the presence of doxycycline for 48 h were probed with anti-Myc antibody. TAO was used as the loading control. (C) Growth curve for TbTim50-Myc (control) and TbTim50-Myc/TbTim50 RNAi (TbTim50 KD) cells grown in the presence of doxycycline. Cell numbers were counted each day for 6 days postinduction. Cells were reinoculated when the parasite number reached 1 × 10^6^ cells/ml. The log of the cumulative cell number was plotted against the number of days postinduction. Standard errors were calculated from three experiments. (D) Effect of TbTim50 KD on mitochondrial ΔΨ. TbTim50-Myc (control) and TbTim50-Myc/TbTim50 RNAi (TbTim50 KD) cells (1 × 10^7^) were harvested at 2 (D2) and 4 (D4) days postinduction and stained with MitoTracker red. Fluorescence intensity was measured with a FACSCalibur (Becton, Dickinson) analytical flow cytometer using absorption at 578 nm and emission at 599 nm. FlowJo software was used to analyze the results. (E) Quantitation of the fluorescence intensity from triplicate samples was performed. Fluorescence intensity of the control was considered 100%. (F) Change in the CL content in BF T. brucei due to Tim50 depletion. CL levels were measured in the cell lysate of control and Tim50 KD cells, as described in Materials and methods. Values shown are means ± standard errors from triplicate samples. Significance values were calculated by *t* test and are indicated by asterisks (**, *P *< 0.01).

To understand the function of TbTim50 in the BF, we developed a TbTim50 RNA interference (RNAi) cell line using the TbTim50-Myc BF as the parental control. Induction of RNAi by doxycycline showed that the level of TbTim50 transcript was reduced by 75% (Fig. 3A), and a similar reduction of the TbTim50-Myc protein level was observed (Fig. 3B). TbTim50 knockdown (KD) reduced the growth rate of the BF significantly, particularly after 4 days postinduction (Fig. 3C). Within 5 to 6 days postinduction, cell doubling time was increased about 3- to 4-fold in comparison to the parental control. Due to leaky expression of TbTim50 RNAi, uninduced cells also showed growth reduction in comparison to the control (Fig. S3A). However, the growth of induced cells was further reduced compared to that of uninduced cells, particularly after day 6. To analyze the effect of TbTim50 KD on mitochondrial membrane potential (ΔΨ), the control and TbTim50 RNAi cells were grown in the presence of doxycycline for 2 and 4 days and stained with MitoTracker red, which is taken up by mitochondria in a ΔΨ-dependent manner (46). Cells were fixed and analyzed by flow cytometry. Results showed that TbTim50 KD reduced mitochondrial ΔΨ (∼60%) by 2 days postinduction of RNAi (Fig. 3D and E). We did not observe any further reduction of mitochondrial ΔΨ after induction of RNAi for 4 days in the BF. Mitochondrial ΔΨ was significantly reduced in induced TbTim50 RNAi cells compared to that in the uninduced control (Fig. S3B). Therefore, as in the PF (demonstrated previously), TbTim50 is required to maintain mitochondrial ΔΨ in the BF. In the BF, mitochondrial ΔΨ is maintained by ATP hydrolysis via the reverse action of ATP synthase. However, using immunoblot analysis, we could not detect any reduction in the levels of this enzyme. As we observed that Tim50 strongly binds with PA and is capable of hydrolyzing PA, a precursor for cardiolipin (CL), we wanted to examine whether TbTim50 knockdown affects the levels of CL in T. brucei. Cardiolipin (CL) is present predominantly in the mitochondrial inner membrane (MIM) and is essential for mitochondrial function (47). During measurement of CL levels in control and TbTim50 RNAi cells grown for 4 days in doxycycline-containing medium, we observed about a 40% reduction of CL levels due to TbTim50 KD (Fig. 3F). This suggests that TbTim50 is linked either to CL synthesis or to instability in the membrane. Loss of CL has been found to be detrimental for mitochondrial ΔΨ in many systems. Therefore, this could be the cause of lower mitochondrial ΔΨ in the TbTim50 KD BF.

10.1128/mBio.01592-21.6 FIG S3 (A) Growth curve for the TbTim50-Myc (control) and TbTim50-Myc/TbTim50 RNAi cells grown in the absence (uninduced) and presence (induced) of doxycycline. Cell growth of the uninduced cells were reduced due to leaky expression of the double-stranded RNA. (B) Fluorescence-activated cell sorting (FACS) analysis of the MitoTracker-stained TbTim50-Myc (control) and TbTim50-Myc/TbTim50 RNAi cells grown in the absence (uninduced) and presence (induced) of doxycycline. (C) Cardiolipin levels were measured in the cell lysate of control (TbTim50-Myc), Tim50 KD (TbTim50-Myc/TbTim50 RNAi), and TbTim50-HA (TbTim50-Myc/TbTim50 KD/TbTim50-HA) cells as described in Materials and Methods. Values shown are means ± standard errors from triplicate samples. Download FIG S3, TIF file, 1.9 MB. Copyright © 2021 Tripathi et al. 2021 Tripathi et al. https://creativecommons.org/licenses/by/4.0/ This content is distributed under the terms of the Creative Commons Attribution 4.0 International license.

### Tim50 knockdown T. brucei could not establish infection in the host.

**FIG 4:**
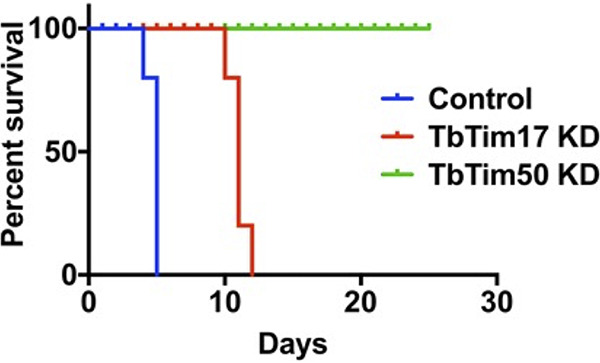
TbTim50 KD T. brucei is unable to establish infection in animals. Survival plot of three groups of mice (5 mice/group) infected with the control, TbTim17 RNAi (TbTim17 KD), or TbTim50 RNAi (TbTim50 KD) BF cells grown for 4 days in the presence of doxycycline. Mice were fed with doxycycline-containing water to continue the RNAi effect. In order to reduce pain and distress, mice were sacrificed when blood parasitemia levels reached >2 × 10^8^/ml, and death was considered 24 h after.

To understand the effect of TbTim50 KD on BF cell growth *in vivo*, we infected three groups of mice with control, TbTim50 KD, and TbTim17 KD BF, respectively. We used TbTim17 KD cells to compare the effect of KD of TbTim50 and TbTim17, as both are Tim proteins and are involved in mitochondrial protein import. We supplemented the drinking water with doxycycline to maintain the induction of RNAi *in vivo*. The group of mice infected with the control BF died within 5 days postinfection, as expected (Fig. 4). The mice infected with the TbTim17 KD BF survived a little longer than those in the control group. The blood parasitemia levels were below the detection levels up to 8 days; after that, the parasite number increased in the blood, and mice died within 12 days (Fig. 4). Surprisingly, mice infected with TbTim50 KD BF did not show any symptoms; parasite levels were below 10^4^/ml of blood, and mice survived more than 3 weeks (Fig. 4). We repeated this experiment three times and observed similar results. Therefore, TbTim50 is crucial for T. brucei cell growth *in vivo*.

### TbTim50 KD hampered cell cycle regulation in the BF.

**FIG 5:**
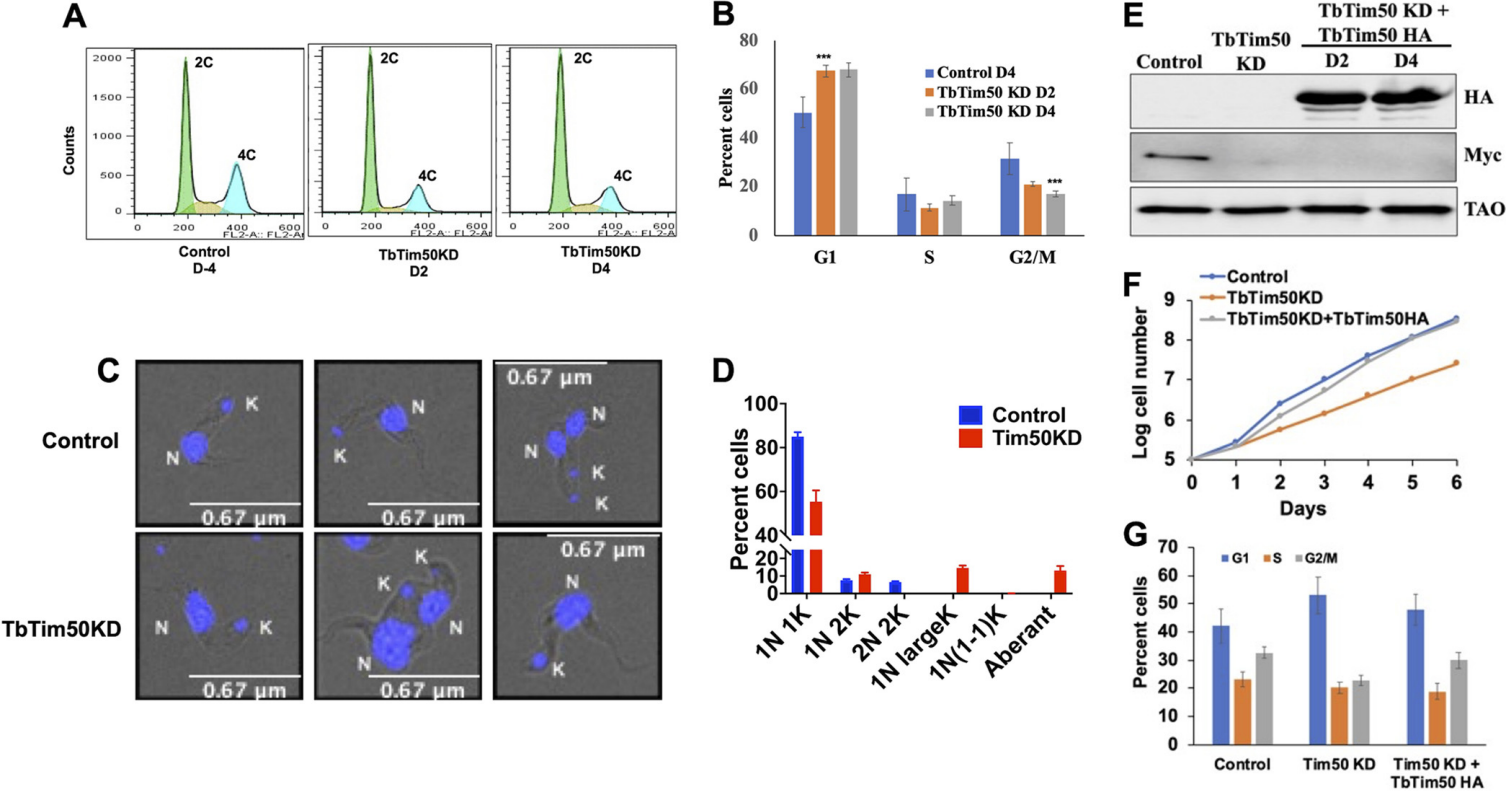
TbTim50 depletion disrupts cell cycle regulation in BF. (A) Fluorescence-activated cell sorting (FACS) profile of propidium iodide-stained control T. brucei cells at day 4 and TbTim50-Myc/TbTim50 RNAi cells at different time points (2 and 4 days) after induction of RNAi. (B) Bar graph representing the percentage of cells in each cell cycle phase (G_1_, S, and G_2_/M), as indicated. (C) Fluorescence microscopy of 4′,6-diamidino-2-phenylindole (DAPI)-stained BF before and after induction of TbTim50 RNAi for 4 days. Nucleus and kinetoplast are marked as K and N, respectively. (D) Quantitation of the numbers of nuclei (N) and kinetoplasts (K) per cell. Values shown are mean percentage of cells ± standard error out of >200 cells of each type. (E) A copy of RNAi-resistant TbTim50-HA synthetic gene was expressed in TbTim50 KD cells as described in Materials and Methods. TbTim50 KD plus TbTim50-HA cells were harvested at day 2 (D2) and 4 (D4) postinduction. Immunoblot analysis of the total cellular proteins from TbTim50-Myc (control), TbTim50-Myc/TbTim50 KD, and TbTim50-Myc/TbTim50 KD/TbTim50-HA cells was performed using antibodies for HA and Myc epitopes and TAO. (F) Cell growth analysis of the control, TbTim50 KD, and TbTim50 KD plus TbTim50 HA cells. (G) Cell cycle analysis of the control, TbTim50 KD, and TbTim50 KD plus TbTim50 HA cells. Cells were stained with propidium iodide and analyzed by FACS. Bar graph representing the percentage of cells in each cell cycle phase (G_1_, S, and G_2_/M) for each cell type.

To elucidate the reason for T. brucei growth inhibition both *in vitro* and *in vivo* due to TbTim50 KD, we analyzed the cell cycle phases. For this purpose, cells were stained with propidium iodide, and the cellular DNA content was measured by fluorescence-activated cell sorting (FACS) analysis. Results showed that TbTim50 KD decreased the population of 4C or G_2_/M phase cells and increased the population of 2C or G_1_ phase cells, suggesting that transition to the mitotic phase is hampered (Fig. 5A). Quantitation of the data from multiple experiments revealed that the increase and the decrease of the G_1_ and G_2_ phase cell populations, respectively, were about 20% (Fig. 5B), which could be accounted for by inhibition of cell growth, as shown in Fig. 3C and Fig. 4. To investigate if the effect on the cell cycle due to TbTim50 KD is reversible, we expressed an RNAi-resistant copy of TbTim50-hemagglutinin (HA) in TbTim50 RNAi cells. Immunoblot analysis showed that TbTim50-HA is overexpressed in TbTim50 RNAi cells, while TbTim50-Myc levels were undetected (Fig. 5E). We also found that cell growth inhibition due to TbTim50 RNAi was reversed due to expression of the RNAi-resistant copy of TbTim50-HA (Fig. 5F). These TbTim50 RNAi cells also showed a decrease in the G_1_ population and an increase in the G_2_/M population in comparison to those of TbTim50 KD T. brucei (Fig. 5G). Furthermore, TbTim50 overexpression raised the CL levels about 20% in comparison to TbTim50 RNAi cells (Fig. S3C). These results clearly showed that the phenotypes we observed were due to loss of the TbTim50 levels in the BF.

**FIG 6:**
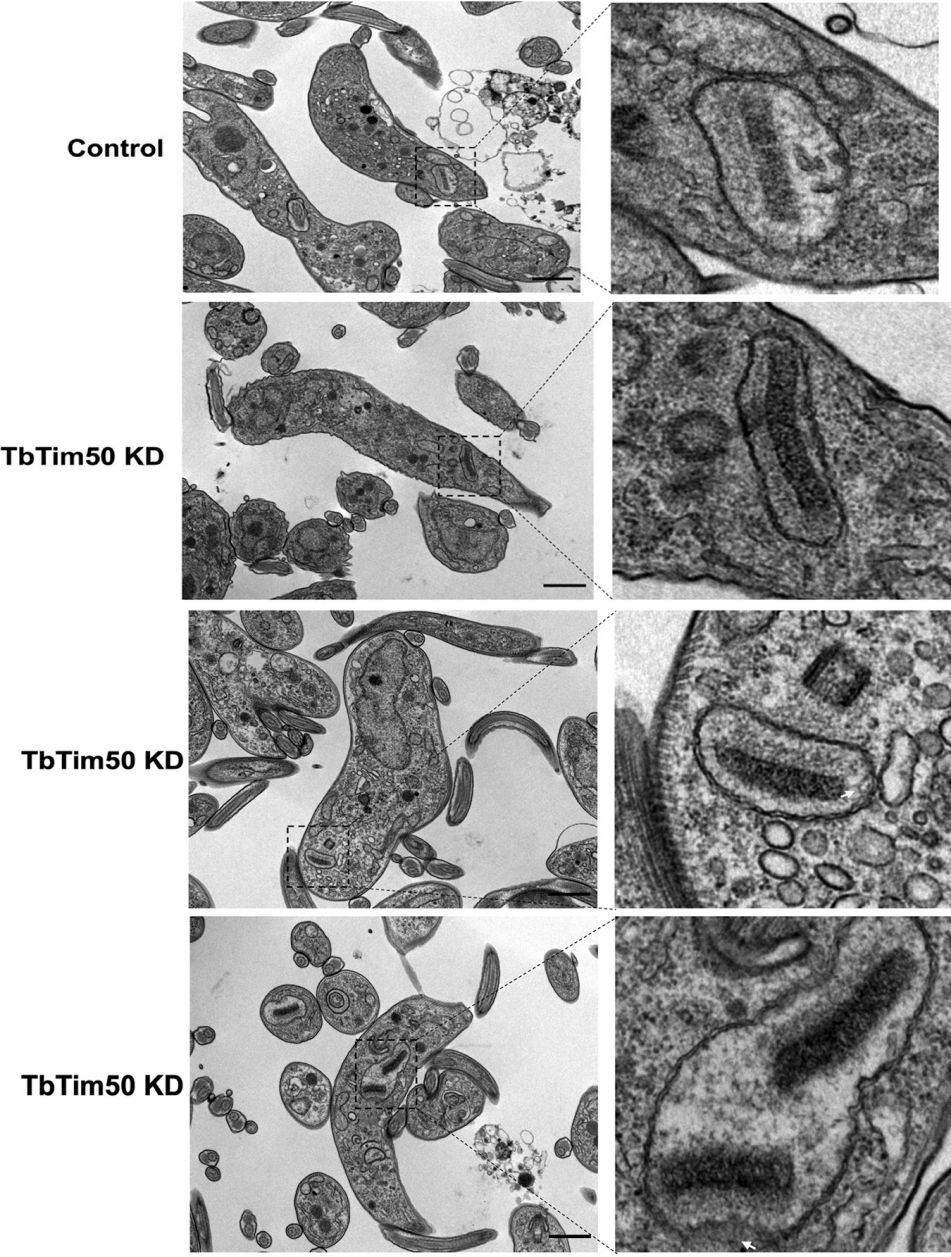
Effect of TbTim50 KD on kDNA structure. Thin-section electron micrographs of the control and TbTim50 KD cells at 4 days postinduction. Representative pictures are shown. The regions in the box containing kDNA in mitochondria are magnified as indicated by the dashed lines. Bars, 1 μm (lower-magnification images) and 500 nm (higher-magnification images). White arrows indicate changes in membrane structure.

During the cell cycle in trypanosomatids, kinetoplast (K) division occurs first, along with the basal body and flagellar duplication, followed by nuclear (N) division and cytokinesis (11, 12). Therefore, in the G_1_ and S phases, each cell possesses 1N1K; G_2_ and S phase cells have 1N2K; and 2N2K cells are mitotic/postmitotic (9). By counting the numbers of N and K per cell from >200 cells each from the control and TbTim50 KD groups, we noticed significant differences in the sizes and numbers of N and K in TbTim50 KD cells. Compared to the control, TbTim50 KD cells had larger K and N, and often K and N were segregated asymmetrically (Fig. 5C and Fig. S4); we noticed that the proportion of 2N2K cells decreased significantly (Fig. 5D). In contrast, 1N large-K, larger N and K, and abnormal cells (no N but K) were accumulated. These results showed that TbTim50 KD cells may have uncontrolled replication or defective segregation of kDNA and thus create larger K. Analysis of T. brucei ultrastructure using electron microscopy (EM) revealed that the kDNAs appear more electron dense on average in TbTim50 RNAi cells than in control cells (Fig. 6). All EM pictures were equally adjusted for contrast, and we found that kDNAs in TbTim50 KD cells were about 30% denser than the wild type. However, during quantitation, we found many outliers, which could be because the cells were not at the same stage of replication. Overall, these results indicate that replication of kDNA likely continued without segregation due to TbTim50 KD in the BF. We also noticed some changes in the inner membrane structure due to TbTim50 RNAi (indicated by arrows in Fig. 6). We believe that this could be due to reduction of CL levels in these cells.

10.1128/mBio.01592-21.7 FIG S4 Fluorescence microscopy of 4′,6-diamidino-2-phenylindole (DAPI)-stained BF before (A) and after (B) induction of TbTim50 RNAi for 4 days. Cells containing 1K1N, 2K1N, 2K2N, larger kinetoplasts (K), and abnormal K are marked with red, yellow, green, black, and purple asterisks, respectively. Download FIG S4, TIF file, 2.8 MB. Copyright © 2021 Tripathi et al. 2021 Tripathi et al. https://creativecommons.org/licenses/by/4.0/ This content is distributed under the terms of the Creative Commons Attribution 4.0 International license.

### TbTim50-depleted cells have altered morphology, increased AMPK phosphorylation, and increased expression levels of stumpy-specific transcripts.

**FIG 7:**
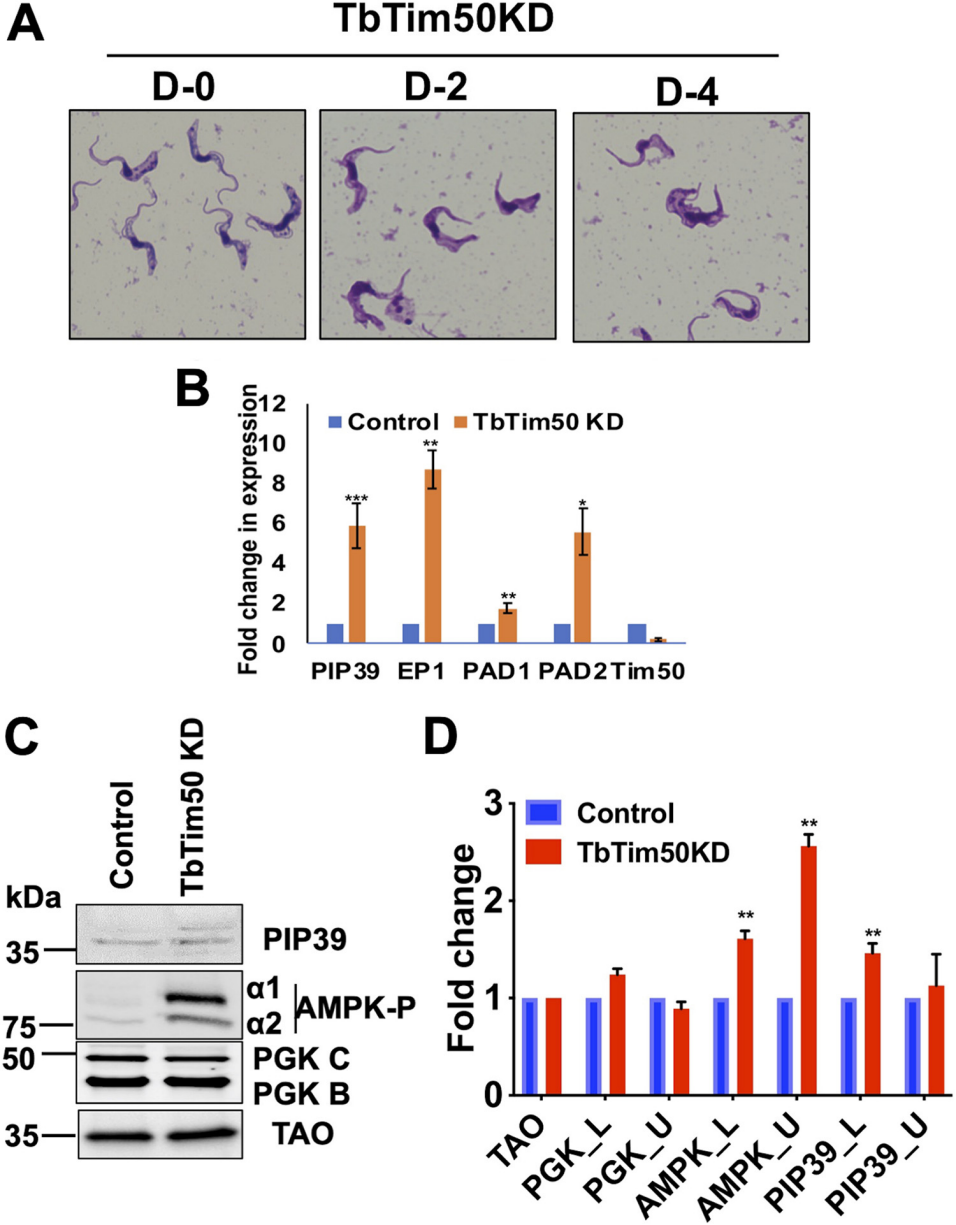
TbTim50 KD altered cell morphology, increased AMPK phosphorylation, and upregulated expression of the ST-specific transcripts. (A) Giemsa-stained TbTim50 RNAi cells at days 0, 2, and 4 after induction with doxycycline. (B) Quantitative RT-PCR analysis of the EP1, PAD1, PAD2, PIP39, and TbTim50 transcripts in control and TbTim50 RNAi T. brucei BF cells grown for 2 days in the presence of doxycycline. Telomerase reverse transcriptase (TERT) was used as the internal control. Relative levels of the marker transcripts compared to those in the parental control were plotted. Averages and standard errors were calculated from three experiments. (C) Immunoblot analysis of total proteins from the control and TbTim50 KD cells harvested after 4 days postinduction using different antibodies against PIP39, phospho-AMPK, PGK, and TAO as probes. (D) Densitometric analysis of the protein bands for PIP39_upper (PIP39_U), PIP39_lower (Pip30_L), AMPKα1P, AMPKα2P, PGK_B, and PGK_C after normalization with that for TAO. Values shown are mean fold increase ± standard error in TbTim50 KD in comparison to the control from triplicate experiments. Significance values were calculated by *t* test and are indicated by asterisks as follows: **, *P* < 0.05; *** *P* < 0.01.

Examining Giemsa-stained cells under a 100× objective, we found that the LS BF cells gradually appeared thicker and more stumpy after induction of TbTim50 RNAi (Fig. 7A). This cell line was derived from the SM427 T. brucei BF, which is a laboratory-adapted monomorphic cell line that loses the capacity to transform to the ST form with increasing cell density (5, 6). As we observed that TbTim50 KD induced a change in morphology in these monomorphic cells, we performed reverse transcription-PCR (RT-PCR) analysis for several stumpy-specific transcripts. Interestingly, we noticed severalfold upregulation of the transcript levels for PIP39 and proteins associated with differentiation (PAD1 and PAD2) in cells where TbTim50 transcript levels were reduced by RNAi (Fig. 7B). Interestingly, the transcript levels of EP1 procyclin was also increased. These results suggest that TbTim50 KD inhibits the cell division that likely triggers expression of these marker genes. It has been shown that monomorphic 427 T. brucei could be transformed to an ST-like form when incubated with AMP or cell-permeable AMP analogue, and transformation of the pleomorphic SL to ST BF by SIF is mediated via AMPK phosphorylation (5, 6). Therefore, we investigated whether AMPK phosphorylation is altered due to TbTim50 KD. Immunoblot analysis indeed showed that levels of phosphorylated AMPK, both α1 and α2, were upregulated due to TbTim50 KD (Fig. 7C and D). In contrast, the levels of PGK and TAO were unchanged. Previously, we showed that TbTim50 KD in the PF increased AMPK phosphorylation and increased the levels of PIP39 protein severalfold (36). Although we observed a significant upregulation of AMPK phosphorylation and an upregulation of PIP39 transcript levels in the TbTim50 KD BF, PIP39 protein levels were increased minimally. PIP39 antibody recognized a pair of bands of expected sizes. The upper band could be the phosphorylated PIP39. The discrepancy between PIP39 transcript and protein levels is likely because PIP39 protein expression is regulated further, at the stage of either protein translation or stability in the monomorphic strain. Overall, we observed that TbTim50 KD disrupts cell cycle regulation, possibly by inhibition of kDNA division and induced expression of some stage-specific transcripts in the BF.

### Global proteomics analysis revealed downregulation of certain cellular functions due to TbTim50 KD.

**FIG 8:**
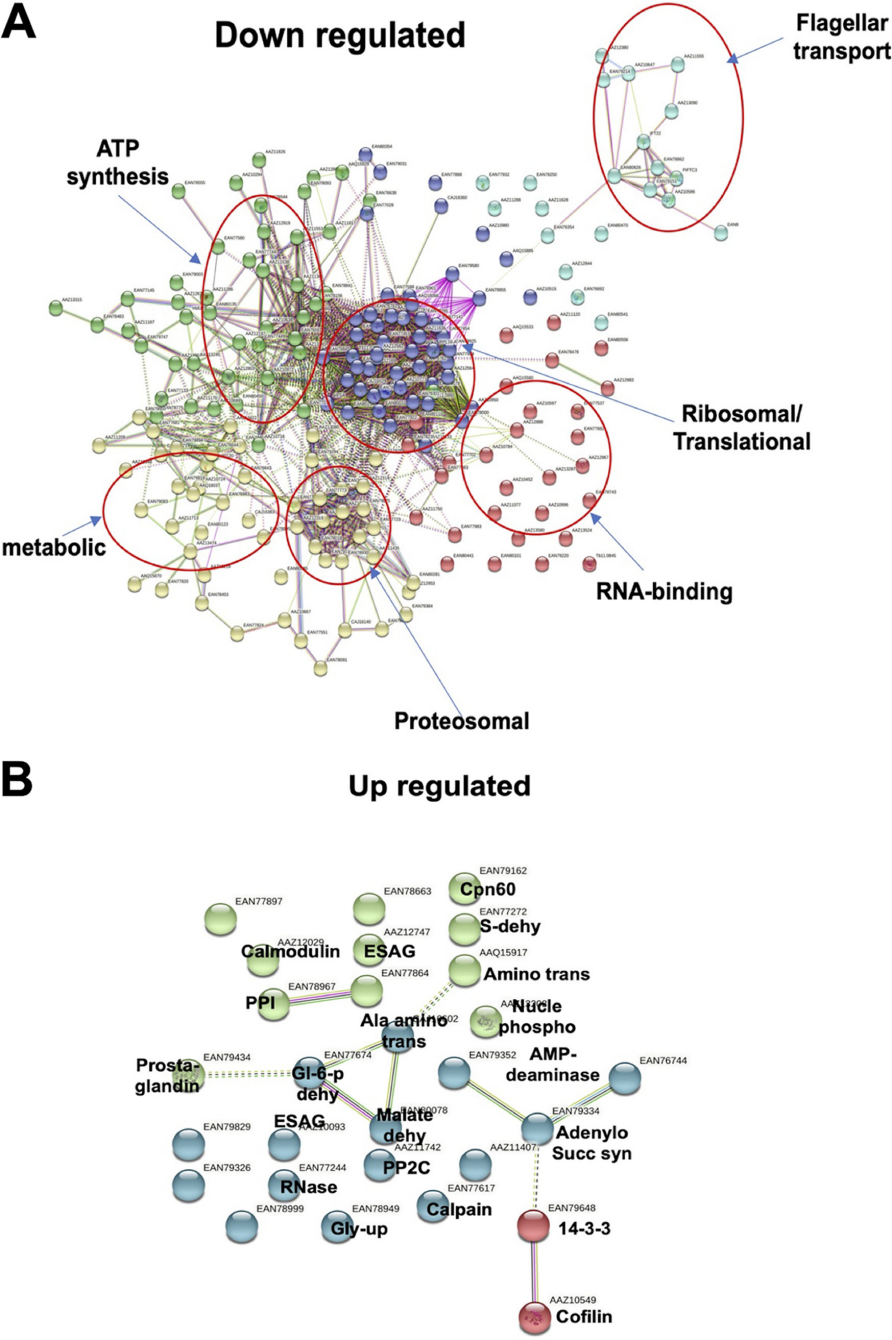
Comparative proteomics analyses and validation of the proteomic results. The semiquantitative proteomics analysis was performed to compare the proteomes in the control and TbTim50 KD cells. Statistically significant downregulated and upregulated proteins from three biological replicates were used for STRING analysis, as described in Materials and Methods. (A and B) Interaction map of proteins significantly downregulated (A) and upregulated (B) due to TbTim50 KD. Clusters of downregulated proteins are circled and labeled according to the gene ontology (GO) term. Upregulated proteins were less clustered, and individual proteins are labeled as identified.

To understand the effect of TbTim50 KD on total cellular proteomes, semiquantitative proteomics analyses were performed using control and TbTim50 RNAi cells grown for 2 and 4 days in the presence of doxycycline. Overall, we found more proteins were downregulated (<0.5-fold) than upregulated (>1.5-fold) in TbTim50 KD cells. From the day 2 sample, we observed that 130 proteins were downregulated and 51 were upregulated (Data Set S1). Similarly, from the day 4 sample, 57 were upregulated and 377 were downregulated (Data Set S2). After overlapping these results, we found that 58 proteins were common in both samples that were altered due to TbTim50 KD (Fig. S5A). Clustering these proteins according to the functional terms revealed the presence of ribosomal (27%), translational factors (12%), RNA binding (12%), cytoskeletal (10%), proteasomal (9%), chaperones (8%), developmental regulation (7%), metabolic (5%), and stress-regulated (3%) proteins (Fig. S5B). Next, we separated the list of upregulated and downregulated proteins and performed STRING analysis (Fig. 8A). From these analyses, we found that downregulated proteins were clustered into different groups, which include (i) ribosomal proteins and translation factors, (ii) flagellar transport proteins, (iii) RNA binding proteins, (iv) proteasome subunits, (v) metabolic enzymes, and (vi) ATP-synthase subunits. Downregulation of many ribosomal proteins and translational factors indicated that protein synthesis was inhibited, which is not unexpected, as we saw that AMPK phosphorylation was increased due to TbTim50 KD. It is known that activated AMPK stimulates catabolic processes and inhibits anabolic processes like protein synthesis (48). Furthermore, we found downregulation of several proteasome subunits that are known to be linked with cell cycle regulation in T. brucei (49). In particular, the mitochondrial heat shock locus ATPase (HslVU) complex, known to play roles in kDNA replication/segregation (50, 51), was downregulated >4-fold (*P* < 0.005) in TbTim50 KD cells. We found that several subunits of the ATP synthase (complex V) complex were downregulated, which could be due to loss of CL or vice versa. Alteration of the levels of the flagellar transport proteins and metabolic enzymes are also indicative of cellular stress/adaptation due to TbTim50 KD. In contrast to downregulated proteins, upregulated proteins were less clustered (Fig. 8B). These include Ca signaling proteins, such as calmodulin, calpain, PP2c, and PP1. Levels of several metabolic enzymes, such as glucose-6-phosphate dehydrogenase, malate dehydrogenase, alanine amino transferase, and glycerol uptake protein were increased. Upregulation of AMP-deaminase, adenylosuccinate synthase, and nucleotide phosphotransferase was indicative of the cellular demand for nucleotides, likely for continuation of kDNA replication. In addition, we noticed some upregulation of several expression site-associated gene (ESAG)-related proteins and of prostaglandin synthase. Overall, the information gathered from proteomics analyses was correlated with the phenotype of the TbTim50 KD T. brucei.

10.1128/mBio.01592-21.1 DATA SET S1 Downregulated and upregulated proteins due to TbTim50 knockdown (KD) at day 2 postinduction. Label-free quantitation of total proteomes in the control (parental) and TbTim50 RNA interference (RNAi) cells grown for 2 days in the presence of doxycycline. Downregulated (red) and upregulated (green) proteins are highlighted. Download Data Set S1, XLSX file, 0.04 MB. Copyright © 2021 Tripathi et al. 2021 Tripathi et al. https://creativecommons.org/licenses/by/4.0/ This content is distributed under the terms of the Creative Commons Attribution 4.0 International license.

10.1128/mBio.01592-21.2 DATA SET S2 Downregulated and upregulated proteins due to TbTim50 KD at day 4 postinduction. Label-free quantitation of total proteomes in the control (parental) and TbTim50 RNAi cells grown for 4 days in the presence of doxycycline. Downregulated (red) and upregulated (green) proteins are shown. Download Data Set S2, XLSX file, 0.05 MB. Copyright © 2021 Tripathi et al. 2021 Tripathi et al. https://creativecommons.org/licenses/by/4.0/ This content is distributed under the terms of the Creative Commons Attribution 4.0 International license.

10.1128/mBio.01592-21.8 FIG S5 Changes in BF proteomes due to TbTim50 KD. (A) Semiquantitative proteomics analyses were performed using control and TbTim50 RNAi cells grown for 2 and 4 days in the presence of doxycycline. Overlap of significantly altered proteins identified 58 common proteins. The levels of these proteins were changed in cells at 2 (D2) and 4 (D4) days after RNAi. (B) The common proteins were classified according to their functional terms and are presented in a pie chart. Download FIG S5, TIF file, 2.8 MB. Copyright © 2021 Tripathi et al. 2021 Tripathi et al. https://creativecommons.org/licenses/by/4.0/ This content is distributed under the terms of the Creative Commons Attribution 4.0 International license.

### TbTim50 depletion reduced levels of mitochondrial HslVU.

**FIG 9:**
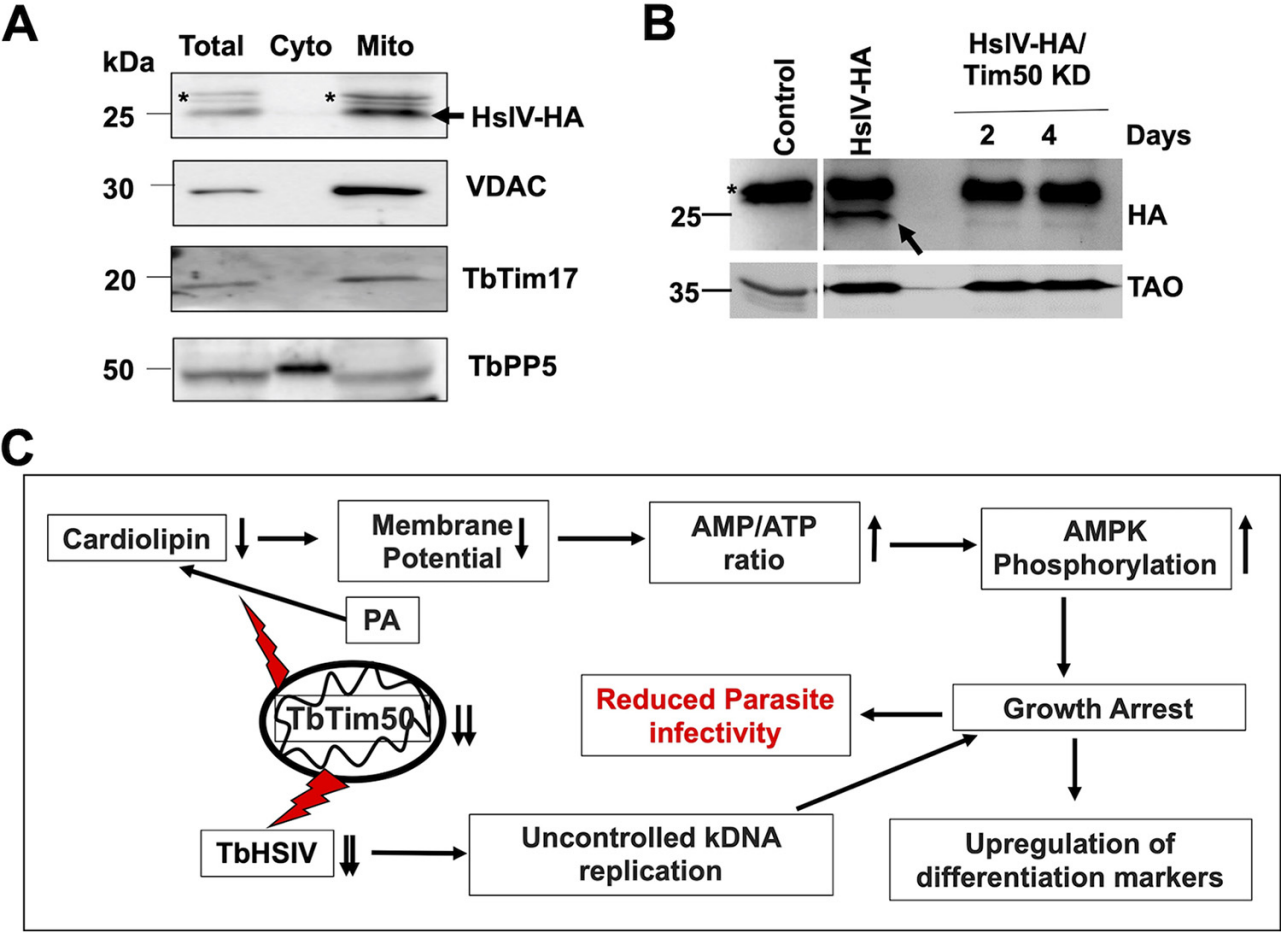
(A) *In situ*-tagged TbHslV-HA is expressed and localized in the mitochondria. Immunoblot analysis of the total, cytosolic (Cyto), and mitochondrial (Mito) fractions from TbHslV-HA cells were probed with anti-HA, VDAC, TbTim17, and TbPP5 antibodies. Nonspecific bands are marked with an asterisk (*), and the position of the specific bands for HslV-HA is indicated by an arrow. (B) The TbHslV-HA cells were further transfected with the TbTim50 RNAi construct to develop a stable TbHslV-HA/TbTim50 KD cell line. Immunoblot analysis of total proteins from the parental control, TbHslV-HA, and TbHslV-HA/TbTim50 KD cells, grown in the presence of doxycycline for 2 and 4 days using anti-HA antibody. The position of the nonspecific bands is marked by an asterisk (*), and the specific bands for HslV-HA are indicated by an arrow. TAO was used as the loading control. (C) The working model of TbTim50 function in the BF. TbTim50 is critical to maintain CL levels in mitochondria. Thus, depletion of TbTim50 hampered mitochondrial ΔΨ, created an energy crisis, and increased AMPK phosphorylation that inhibits cell growth and triggers some differentiation processes. TbTim50 also is required for TbHslVU functions to regulate the kDNA replication and cell cycle progression. The growth-arrested cells generated due to TbTim50 KD are cleared rapidly from the bloodstream in a mammalian host. Therefore, the TbTim50-depleted parasite could not establish infection.

From our proteomic analysis, we observed that mitochondrial HsIVU subunits were consistently downregulated >4-fold in all biological replicates. HslVU is a prokaryotic proteasome complex found in mitochondria in certain eukaryotic species, including trypanosomatids (51–53). T. brucei and related parasites thus have both mitochondrial HslVU and cytosolic 26S proteasome complexes. Interestingly, the HslVU complex has not been found in animal or human mitochondria. The HslVU complex consists of the catalytic subunit HslV and the regulatory subunit HslU (51, 52). The HslV subunit forms a dodecameric homo-oligomer, which is shaped like double donuts stacked on top of each other. The holo-regions on both sides are blocked by homohexameric HslU. HslU binds substrate proteins, unfolds them by ATP hydrolysis, and transfers to the catalytic core for degradation. T. brucei has two HslU subunits, U1 and U2; TbHslU1 is only able to activate TbHslV (52). Multiple reports have shown that TbHslVU plays a role in kDNA replication and segregation during the cell cycle in T. brucei (50–53). TbHslV KD caused T. brucei to have larger K and N and asymmetrically divided kDNA, similar to what we found in TbTim50 KD cells. It has been shown that PIF2 helicase, which is needed for maxicircle replication, is degraded by HslVU to regulate kDNA replication and segregation (53). Therefore, we selected TbHslV to further validate our proteomics results. For this purpose, we *in situ*-tagged TbHslV at the C terminus with 3×HA. The selected cell line showed expression of the expected-size HA-tagged protein in the mitochondrial fraction (Fig. 9A). These cells were further transfected with the TbTim50 RNAi construct to observe the effect of TbTim50 KD on the levels of TbHslV. We indeed found that the TbHslV levels were reduced significantly due to depletion of TbTim50 (Fig. 9B). To visualize the reduced levels of TbHslV-HA, we had to increase the exposure time, which created a thicker upper band in Fig. 9B in comparison to that in Fig. 9A. A full-gel picture is shown in the supplemental material (Fig. S6). Therefore, our results demonstrated that reduction of the mitochondrial proteasome subunit levels is linked with the phenotype seen in TbTim50-depleted parasites.

10.1128/mBio.01592-21.9 FIG S6 Effect of TbTim50 KD on TbHslV expression levels. (A) Immunoblot analysis of total proteins from SM BF, TbHslV-HA, and TbHslV-HA/TbTim50 KD cells. TbHslV-HA/TbTim50 KD cells were grown in the presence of doxycycline, and samples were harvested at days 2 and 4 postinduction. (B) The immunoblot for the same samples was exposed longer to visualize the TbHslV protein band in TbTim50 KD samples. Specific bands for TbHslV are marked by an asterisk (*). Download FIG S6, TIF file, 2.6 MB. Copyright © 2021 Tripathi et al. 2021 Tripathi et al. https://creativecommons.org/licenses/by/4.0/ This content is distributed under the terms of the Creative Commons Attribution 4.0 International license.

Overall, our finding reveals that TbTim50 has a two-pronged function in mitochondria. It is involved in maintaining CL levels in the MIM. Thus, depletion of TbTim50 reduced mitochondrial ΔΨ and cellular ATP levels, as found in CL synthase KD in the BF (54). Cellular energy crisis activates AMPK by phosphorylation and induces changes in cell morphology and many cellular activities. This form is rapidly cleared by host immunity and could not establish infection. TbTim50 is either directly or indirectly involved in regulation of HslVU; therefore, TbTim50 KD caused unregulated replication of kDNA and hampered cell division, leading to growth inhibition both *in vitro* and *in vivo* (Fig. 9C).

## DISCUSSION

Here, we investigated the role of TbTim50 in the T. brucei BF. As in the PF, TbTim50 is required to maintain mitochondrial ΔΨ. TbTim50 is also critical for BF survival both *in vitro* and *in vivo*. Maintenance of mitochondrial ΔΨ is a conserved function for Tim50 among different species (55, 56). In yeast, it has been shown that Tim50 interacts with Tim23 and with the MIM via CL interaction to close the TIM23 channel in the absence of preproteins (57), thus maintaining the MIM permeability barrier. Our current understanding indicates that TbTim50 interacts weakly with TbTim17, the counterpart of ScTim23 in T. brucei (14, 25). Therefore, it is not clear whether that interaction is required to maintain mitochondrial ΔΨ. However, we found that CL levels are reduced significantly due to TbTim50 KD. As CL, the specialized lipid in the MIM, plays a critical role in membrane integrity, it could be conceivable that the loss of CL is also a cause of reduced mitochondrial ΔΨ. In the BF, mitochondrial ΔΨ is primarily maintained by ATP synthase, acting in reverse orientation (58). Although we did not notice any reduction of ATP-synthase α or β subunits by immunoblot analysis, semiquantitative proteomics analysis showed reductions in the levels of α, β, and γ subunits of the ATPase complex (see Data Sets S1 and S2 in the supplemental material). Loss of CL may hamper ATPase assembly and function in the mitochondria, which could be an additive effect for leaky MIM in the TbTim50 KD BF. It has been shown recently that the KD of CL synthase (CLs) in the T. brucei BF reduced cellular ATP levels and mitochondrial ΔΨ; as a result, cell growth was reduced (54). We found a similar situation in the TbTim50 KD BF due to reduction of CL.

CL is synthesized in the mitochondria from PA and CTP in a multistep process (59, 60). The last step for CL synthesis in T. brucei has been shown to be similar to that in bacteria rather than to that in fungi and mammals (61, 62). T. brucei CL synthase (TbCLs) utilizes two molecules of phosphatidylglycerol (PG) to make CL, whereas CLs in other eukaryotes utilizes PG and CDP-diacylglycerol (CDP-DAG). Interestingly, dephosphorylation of PGP by a phosphatase generates PG. Human protein tyrosyl phosphatase localized in mitochondria (PTPMT1) and yeast GEP4 acts as the PGP phosphatase (63, 64). In fact, GEP4 is a member of the HAD phosphatase family (64). Therefore, involvement of TbTim50, which is also an HAD phosphatase, in CL synthesis is highly probable. Furthermore, CL stability primarily depends on its association with membrane proteins (65). Therefore, TbTim50 loss may increase CL degradation. CL oxidation also increases its degradation (66). In this regard, we have shown that TbTim50 KD increased reactive oxygen species (ROS) production (33, 34), and therefore it may also contribute to CL degradation. Whether TbTim50 participates in CL synthesis or the loss of TbTim50 binding with CL in TbTim50 KD parasites reduces the stability of CL requires further investigation.

Other than the effect on mitochondrial ΔΨ, we observed that TbTim50 KD increased the levels of AMPK phosphorylation in the BF, suggesting an energy crisis due to TbTim50 KD. In the BF, ATP is not synthesized in mitochondria; instead, it is produced solely by glycolysis (67). Mitochondrial activities, such as reoxidation of the reducing equivalents generated during glycolysis via mitochondrial glycerol-3-phosphate dehydrogenase (GPDH) and trypanosome alternative oxidase (TAO), are indeed linked for a continuous flow of glucose oxidation to produce sufficient energy (68, 69). Although we did not observe any significant reduction in the steady-state levels of TAO in our immunoblot analysis, we found GPDH in the list of downregulated proteins identified by proteomics analysis. Therefore, it may be estimated that the loss of mitochondrial membrane integrity hampered the electron transfer processes and slowed down glycolysis; thus, ATP levels were reduced. A similar situation has been reported for TbCLs KD (54). In addition, we found upregulation of the glycerol uptake protein and of a number of amino acid metabolic enzymes in TbTim50 KD cells by proteomics analysis, which also could be an attempt for the cellular adaptation process.

Most interestingly, we found that TbTim50 KD deregulates the cell cycle controls in the BF. The number of cells in the G_1_/S phase increased, and thus the proportion of cells at the G_2_/M phase was reduced significantly due to depletion of TbTim50 levels in the BF. It is known that treatment of LS BF cells with AMP or cell-permeable AMP analogue activates AMPK by phosphorylation, which triggers cells to differentiate into nondividing ST form (5, 6). The laboratory-adapted monomorphic strains lose their capacity to transform to the ST BF. Upon treatment with AMP or AMP analogues, these strains assume an ST-like morphology (70). Interestingly, we noticed that TbTim50 KD changes the monomorphic LS BF morphology. These changes were also associated with upregulation of ST-specific transcripts, including those of PIP39, PAD1, and PAD2. Although PIP39 transcript levels were upregulated 5-fold, PIP39 protein levels were minimally increased in this cell line due to TbTim50 KD and possibly due to additional blockage at the level of translation or PIP39 protein stability. Together, these results show that a sudden drop in cellular energy due to mitochondrial disfunction in TbTim50-depleted cells triggers a differentiation signal; however, it was likely not completed due to a certain roadblock in the monomorphic BF T. brucei.

Comparative proteomics analyses of the control and TbTim50 KD cells and further validation by biochemical analysis helped us to clarify why the LS BF cells were arrested at the G_1_ phase in the cell cycle. Network analysis of the proteins downregulated due to TbTim50 depletion identified a cluster of proteasome subunits. These include mitochondrial HslVU complex subunits; cytosolic 26S proteasome subunits, i.e., PA26, subunit α; and non-ATPase subunits 1, 2, 3, 4, 5, 9, and 11. Both mitochondrial and cytosolic proteasomes are known to play roles in regulation of the cell cycle in T. brucei (49). The HslVU complex, which is unique to mitochondria in trypanosomatids and is not found in their mammalian hosts, is of particular interest (50–53). We clearly see a downregulation of TbHslV due to TbTim50 KD not only by proteomics analysis, but also experimentally. In addition, we observed that TbTim50 KD caused a similar phenotype of kDNA overreplication and/or a defect in segregation like that reported due to KD of the TbHslVU (51, 53). Therefore, we conclude that reduction in the levels of TbHslVU complex due to TbTim50 depletion is the cause for cell cycle dysregulation and reduced cell growth. It is likely that the reduced levels of HslVU continued the replication and abnormal segregation of the kDNA. Kinetoplast division and segregation are the preceding steps for the downstream events such as mitosis and cytokinesis in T. brucei. Therefore, defects in kDNA segregation due to a reduction in the levels of HslVU may relay the signal to cytosolic proteasomal subunits that are responsible to regulate the levels of specific cyclins and other cell cycle regulatory proteins. Therefore, TbTim50 KD caused larger K and N and inhibition of cell division. As HslVU requires ATP for its function, it could be imagined that loss of energy in TbTim50 KD cells may cause this complex to become nonfunctional and unstable. However, reduction of HslVU was not reported due to TbCLs KD, which produces a similar energy crisis (54). Therefore, it is possible that TbTim50 has a direct role in regulation of the TbHslVU complex, which could be by posttranslational modification, i.e., phosphorylation/dephosphorylation. Recent data on humans and yeast showed that Tim50 is the substrate for mitochondrial phosphatase Pptc7 and that the phosphorylated form of Tim50 has higher activity for mitochondrial protein import (71). Phosphoproteomics data sets (TriTrypDB) showed that TbTim50 is phosphorylated at the Thr72 residue and therefore, has the potential to be regulated by kinases/phosphatases.

Overall, we showed that TbTim50 is critical for the function of the mitochondrion in T. brucei BF and is essential for parasite survival. It is crucial to elucidate how TbTim50 is linked to maintaining the levels of CL and TbHslVU in mitochondria to sustain BF cell growth both *in vitro* and *in vivo*.

## MATERIALS AND METHODS

### T. brucei cell culture and transgenic cell lines.

The BF single marker (SM) cell line of Trypanosoma brucei 427 was cultured in HMI-9 medium supplemented with 10% fetal bovine serum and 2.5 μg/ml G418 at 37°C in a CO_2_ incubator (5% saturation) (72). Transfection of the SM BF with different constructs was performed using an Amaxa Nucleofector kit (Lonza, Cologne, Germany) as described previously (TrypsRU Home; https://tryps.rockefeller.edu/).

### Phosphatidic acid phosphatase activity assay.

Phosphatidic acid phosphatase (PAP) activity was measured in a reaction mixture contained 50 mM Tris-HCl buffer (pH 7.5), 1 mM MgCl_2_, and 0.4 mM 1,2-dioctanoyl-*sn*-glycero-3-phosphate (DiC8 PA; Avanti Polar Lipids) in a total volume of 50 μl. Reactions were carried out in triplicate by the addition of recombinant proteins (50 to 250 ng) at 30°C for 30 min. The reaction was terminated by the addition of 100 μl of PiBlue reagent (BioAssay Systems), and the color was allowed to develop at room temperature for 30 min. Absorbance was measured with a spectrophotometer (Bio-Rad) at 620 nm. The amount of phosphate produced was quantified from a standard curve using potassium phosphate and PiBlue reagent. Enzymatic activity was expressed as pmol phosphate released per minute.

### Lipid binding assay.

A phosphoinositide binding assay was performed using PIP Strips (Thermo Fisher), which contain 100-pmol spots of various phosphoinositides on a nitrocellulose membrane. The membrane was blocked with 3% fatty acid-free bovine serum albumin (BSA) in Tris-buffered saline with Tween 20 (TBST) buffer (50 mM Tris-HCl [pH 7.4], 150 mM sodium chloride, and 0.1% Tween 20) for 1 h, followed by overnight incubation with 0.5 μg/ml of purified recombinant proteins (GST-TbTim50 wild type and mutants) in blocking solution at 4°C. Purified GST was used in parallel as control. The membrane was washed three times in TBST buffer and probed with anti-GST antibody for 2 h at 25°C. Subsequently, the membrane was washed with TBST buffer three times and incubated with horseradish peroxidase (HRP)-conjugated anti-rabbit IgG. Signal was detected using chemiluminescence.

### Measurements of cardiolipin levels.

Total cardiolipin (CL) in cells was measured by fluorometric assay kit (Abcam) according to the manufacturer’s instructions in triplicate in a 96-well fluorescence black microtiter plate. TbTim50 RNAi and control cells were grown in the presence and absence of doxycycline for 4 days, respectively. Both types of cells (1 × 10^7^) were harvested by centrifugation at 1,500 × *g* for 10 min and washed twice with cold phosphate-buffered saline (PBS). Cell pellets were resuspended in CL assay buffer, and detergent-free lysis of cells was carried out by sonication in ice. Cell lysates were centrifuged at 10,000 × *g* for 10 min at 4°C, and the supernatants were transferred to fresh microcentrifuge tubes. Soluble supernatant equivalent to 50 μg of proteins were used for the assay. Reactions were carried out in a 96-well plate after addition of 50 μl of probe mix to each well in a total volume of 100 μl. Blank reactions for each sample were performed in parallel without addition of the probe mix. The plate was incubated in the dark at room temperature for 5 to 10 min. Absorbance was measured at excitation/emission of 340/480 nm using a fluorescence microplate reader (BIOtech). A standard curve was generated using the calculated amount of cardiolipin supplied in the kit. Cardiolipin levels in the cell lysates were assessed after subtraction of the blank reading from each of the sample readings.

### Animal experiments.

All experimental procedures were carried out in the Animal Care Facility at Meharry Medical College (Nashville, TN), according to the protocol approved by the Institutional Animal Care and Use Committee. BALB/c mice (4 to 6 weeks old) were purchased (Envigo) and kept in cages (1 to 4 mice/cage) on a 12-h daylight cycle at room temperature. Infection was initiated by the intraperitoneal (i.p.) injection of the T. brucei BF (10^6^ cells/kg body weight). The drinking water for both the control and experimental groups was supplemented with doxycycline (1%) and sucrose (5%), 7 days prior to infection. The parasitemia level in infected animals was monitored by parasite count in blood collected by tail snipping on every alternate day. Animal health was monitored frequently during infection. Animals were euthanized if they were clinically moribund (hunched back, ruffled fur coat, hind limb paralysis, weakness) or had a parasite count of ≥5 × 10^8^/ml of blood.

### Other materials and methods.

Materials and methods for crude mitochondrion isolation, membrane potential measurement, cell cycle analysis, quantitative RT-PCR (qRT-PCR), immunoblot analysis, immunofluorescence imaging, expression and purification of the recombinant protein, electron microscopy, mass spectrometry, bioinformatics, homology modeling and molecular docking, densitometry, and statistical analysis are included in the supplemental material (Text S1).
