# Supplementary material for: Trypanosoma brucei Tim50 Possesses PAP Activity and Plays a Critical Role in Cell Cycle Regulation and Parasite Infectivity
Source: mBio. 2021 Sep 14;12(5):e01592-21. doi: 10.1128/mBio.01592-21 (PMC8546626; doi:10.1128/mBio.01592-21)
Supplement: TABLE S1 [file mbio.01592-21-st001.docx]

**Supplementary Table S1.** Primers used in this study

| Name | Sequence (5’---3’) | Length (bp) |
| --- | --- | --- |
| TbTim50RNAi F | AGTCGGATCCGCATAGAGGGGAAAAGAGTGAGG | 33 |
| TbTim50RNAi R | AGTCAAGCTTGGACGAAAAGCAACATAAACGGTG | 33 |
| TbTim50-Myc P1 | GATAGGATCCATGAACCACGATGCCATGTC | 30 |
| TbTim50 (1269)- P2 | GATCCTCGAGTCAGTAATGCGGAGAGTTTTGTC | 33 |
| TbTim50-(703) P3 | GGTCAAGCTTACAAGATCACACTTATATTAGATC | 34 |
| TbTim50-pNAT P4 | GGATCCTCACCTAGGCAGGTCTTCTTCAGA | 30 |
| TbHslV-HA P1 | GATCAAGCTTTGTTGTCATTGGTTTCGCGG | 30 |
| TbHslV-HA P2 | GATCTCTAGACTCGCTAGTTTTCGCCGAC | 29 |
| rGST-TbTim50 F | GATAGGATCCATGAACCACGATGCCATGTC | 30 |
| rGST-TbTim50 R | GATCCTCGAGTCAGTAATGCGGAGAGTTTTGTC | 33 |
| EP1- qRT-PCR F | GAAGGACCAGAAGACAAGGG | 20 |
| EP1- qRT-PCR R | AGGTTCAGGCTCAACTTCGT | 20 |
| TbPAD1-qRT-PCR-F | CAGCGAGATCTCTCCACCAT | 20 |
| TbPAD1-qRT-PCR-R | TGCGATCACGAAGATAGGCT | 20 |
| TbPAD2-qRT-PCR F | GGTCTCGCCTTCCCAGCATT | 20 |
| TbPAD2-qRT-PCR R | TCAGCGTTATCGTCGCAGGT | 20 |
| TbTim50-qRT-PCR F | CCGCCTCCGTCTCGGTTTAT | 20 |
| TbTim50-qRT-PCR R | CCAGGTCCCGACCAAGCAAT | 20 |
| TERTqRT-PCR F | GAGCGTGTGACTTCCGAAGG | 20 |
| TERTqRT-PCR R | AGGAACTGTCACGGAGTTTGC | 21 |
| PIP39qRT-PCR F | TGGAGGTGCAGGTGTTACAA | 20 |
| PIP39qRT-PCR | CGTTTGAGTTCGGGAAAGGG | 20 |
| Tim50D242A, D244A F | GCTGTGTACAAGCGTCTCAGCAAGAGCTAATATAAGTGTGATCTTGTC | 48 |
| Tim50D242A, D244A R | GACAAGATCACACTTATATTAGCTCTTGCTGAGACGCTTGTACACAGC | 48 |
| Tim50D345A, D347A F | GATAGCGACACGAGCCAGGGCCCGACCAAGCAA | 33 |
| Tim50D345A, D347A R | TTGCTTGGTCGGGCCCTGGCTCGTGTCGCTATC | 33 |
